# Tuberculosis risk among people with diabetes mellitus in Sub‐Saharan Africa: A systematic review

**DOI:** 10.1111/tmi.13733

**Published:** 2022-02-28

**Authors:** Ilja Obels, Sandra Ninsiima, Julia A. Critchley, Peijue Huangfu

**Affiliations:** ^1^ Master’s student Biomedical Sciences Radboud University Nijmegen Nijmegen The Netherlands; ^2^ Makerere University College of Health Sciences Kampala Uganda; ^3^ Population Health Research Institute St George’s University of London London UK

**Keywords:** diabetes mellitus, HIV, sub‐Saharan Africa, systematic review, tuberculosis

## Abstract

**Objectives:**

People with diabetes mellitus (DM) have a higher tuberculosis (TB) risk, but the evidence from sub‐Saharan Africa (SSA) was scarce until recently and not included in earlier global summaries. Therefore, this systematic review aims to determine the risk of active TB disease among people with DM in SSA and whether HIV alters this association.

**Methods:**

Medline, Embase, CINAHL, Web of Science, Global Health and African Index Medicus were searched between January 1980 and February 2021. Cohort, case‐control and cross‐sectional studies from SSA, which assessed the association between DM and active TB, were included if adjusted for age. Two researchers independently assessed titles, abstracts, full texts, extracted data and assessed the risk of bias. Estimates for the association between DM and TB were summarised using a random effects meta‐analysis. PROSPERO: CRD42021241743.

**Results:**

Nine eligible studies were identified, which reported on 110,905 people from 5 countries. Individual study odds ratios (OR) of the TB–DM association ranged from 0.88 (95% CI 0.17–4.58) to 10.7 (95% CI 4.5–26). The pooled OR was 2.77 (95% CI 1.90–4.05). High heterogeneity was reduced in sensitivity analysis (from *I* ^2^ = 57% to *I* ^2^ = 6.9%), by excluding one study which ascertained DM by HbA1c. Risk of bias varied widely between studies, especially concerning the way in which DM status was determined.

**Conclusions:**

There is a strong positive association between DM and active TB in SSA. More research is needed to determine whether HIV, a key risk factor for TB in SSA, modifies this relationship.

## INTRODUCTION

Tuberculosis (TB) remains a major global health concern, with 10 million cases and 1.4 million deaths in 2019 [1]. Diabetes mellitus (DM) is known to increase infection risk and severity of many infectious diseases, including TB [2]. It has been estimated that in 2019, 463 million people had DM worldwide [3]. According to population‐based studies, half of these people remained undiagnosed [3]. Especially in sub‐Saharan Africa (SSA), the prevalence of DM is increasing rapidly. By 2045, the number of adults with DM in SSA is projected to increase by 142.9% compared with 2019 [3], due to ageing of the currently young population and rising levels of urbanisation altering traditional lifestyles and diets [4, 5].

Several studies have established that DM increases the risk of active TB (ATB) by 2–3 times, but evidence from SSA is sparse [6, 7, 8, 9, 10]. Previous reviews on the risk of TB in DM have only scarcely included studies from SSA. A review conducted by Al‐Rifai et al. in 2017 included only one study conducted in SSA, and most of the evidence came from high‐income countries [6]. The association between DM and TB could potentially be different in an African setting due to heterogeneity in DM phenotype and presentation, poorer DM management, differences in TB incidence, and in particular, a higher prevalence of HIV. People with HIV have a 27–32 times greater chance to develop ATB than HIV‐negative people, which makes HIV a very important risk factor for TB in SSA [11, 12]. There is little known about the possible effect‐modification of the association between DM and TB by HIV status. In 2017, Bailey et al. published a systematic review on this topic, in which they identified only three eligible studies [13]. No conclusion could be drawn, because some studies suggested that the effect of DM on TB risk might be greater in people with HIV, and other studies suggested the opposite [13]. No strong evidence for any association between TB and DM in SSA was identified in this review.

As far as we are aware, no other systematic review has been conducted on the association between DM and TB in SSA. However, the body of literature on this topic has grown rapidly over the past 5 years since research for the Bailey review was completed. Therefore, the aim of the current systematic review was to determine the risk of ATB among people with DM (either type 1 or type 2, though mainly the latter) in SSA. A sub‐focus was whether HIV modifies this association.

## METHODS

The review protocol was registered in the International Prospective Register of Systematic Reviews (PROSPERO) on the 11^th^ of March 2021 (registration number CRD42021241743).

### Search strategy

We searched Medline (via PubMed), Embase (via Ovid), CINAHL, Web of Science, Global Health (via Ovid) and African Index Medicus for studies published between January 1980 and February 2021. Prior to 1980, DM prevalence in SSA was significantly lower, HIV not yet discovered and TB treatment different; therefore, earlier studies may not be comparable. Search strings included MESH, keyword terms and synonyms for the words ‘’tuberculosis’’, "Diabetes Mellitus" and "Africa" and the names of each sub‐Saharan African country (Appendix 1). In addition to database searches, reference lists of eligible studies, key reviews and conference abstracts of the International Union Against Tuberculosis and Lung Disease conferences from 2016 to 2020 were hand‐searched to identify potentially relevant studies.

### Eligibility criteria

Cohort studies, case‐control studies and cross‐sectional studies that determined the association between DM and TB in SSA were included. Studies were included when they adjusted for at least the confounder age, which is thought to be an important confounder in this association globally, and had a suitable control group [14]. The key exposure was DM, as defined by the individual studies (generally patient reported, abstracted from medical records or diagnosed by blood glucose tests/glycosylated haemoglobin). The main outcome was incident TB disease. Studies were included irrespective of DM type (which was often not reported, but likely mostly DM type 2), TB type (pulmonary and extrapulmonary), and methods used for DM and TB ascertainment. For the sub‐focus on effect‐modification by HIV, an additional inclusion criterion was that the studies stratified the estimate of the association by HIV status, that is, estimating association between TB and DM in people with HIV and those without separately.

We excluded case series, reviews, commentaries and other publications without primary data, studies that could not be obtained from any source (online databases, library request or contacting authors), those not published in English or French and animal studies. Furthermore, studies with predominantly participants below 18 years of age were excluded, because the prevalence of DM is significantly different in children.

### Study selection and data extraction

Titles and abstracts of studies identified with the searches were screened independently by two researchers (IO, SN). Of potentially relevant studies, full texts were obtained which were again screened by these two researchers independently. Any disagreements on eligibility were resolved through discussion or consultation with a third researcher (PH or JAC).

From the studies included, the following data were extracted: study characteristics (author, publication year, study period, study design, country, setting, language, inclusion/exclusion criteria, potential confounders adjusted for), diagnostics used to identify people with TB, DM and HIV, baseline characteristics of participants (sample size, age, sex, body mass index (BMI), fasting blood glucose (or related), HIV status and history, new TB cases, TB/DM history, TB type (pulmonary or extra‐pulmonary), culture positivity, TB symptoms, new DM cases) and outcomes (odds ratios (ORs) or other measures that quantify the association between DM and TB including the number of cases and confidence intervals). When multiple adjustment models were presented, the model which adjusted for most confounders was chosen. As with study selection, data extraction was performed by two researchers independently (IO, SN) and any discrepancies were resolved through discussion or consultation with a third researcher (PH or JAC).

### Choice of effect measure

Some studies reported several estimates for the association, for different timepoints over the course of TB treatment, or different diagnostics for DM. For consistency with other studies, we included estimates at enrolment (compared with those at follow‐up). Additionally, the estimate for the most reliable test for DM or TB was included. This meant that in the main analysis, glycosylated haemoglobin (HbA1c), oral glucose tolerance test (OGTT) and fasting blood glucose (FBG) measurements in venous blood were prioritised over random blood glucose measurements and measurements in capillary blood [15]. Furthermore, when the estimates were stratified by HIV status, the estimate of the association among HIV‐negative participants was included, since the main analysis did not aim to assess the effect of HIV on the association. The impact of these choices on overall results was assessed in sensitivity analyses (Appendix 3). Furthermore, sensitivity analyses excluding studies that used only patient self‐report to classify DM status or that had no microbiological confirmation of TB diagnosis were performed to assess the influence of inclusion of these studies on estimates of the association between TB and DM.

### Statistical analysis

To summarise evidence, study outcomes were pooled statistically. Meta‐analysis for TB risk was performed using a random effects method, because heterogeneity between the studies included was expected. To obtain a weighted average, a Mantel‐Haenszel analysis was performed. Subsequently, statistical heterogeneity was assessed using a chi‐squared test and the degree of heterogeneity was determined using the *I* ^2^ statistic. Finally, a funnel plot was made to detect publication bias. Because Cochrane recommends not to use Egger's test for small study effects for less than 10 studies, Egger's test was not performed [16]. All statistical analyses were performed in STATA 16 [17].

### Risk of bias assessment

To assess the quality and risk of bias of the studies included, we applied the validated Newcastle‐Ottawa scale for cohort and case‐control studies (Appendix 2) [18]. Because this scale was not designed to assess cross‐sectional studies, the Quality assessment tool for observational, cohort and cross‐sectional studies of the National Institute of Health was applied for cross‐sectional studies (Appendix 2) [19]. Risk of bias assessment was conducted by two researchers independently (IO, PH). Disagreements were resolved by consultation with a third researcher (JAC), as with inclusion and extraction.

Bias arising from the ascertainment of DM was considered low if an internationally recognised method of diagnosing DM was used in the study, such as measuring plasma glucose or HbA1c, intermediate if less accurate capillary measurements were used and high if DM status was self‐reported only. Bias from the ascertainment of TB was judged low if TB was confirmed by culture or XPERT and high if based on symptoms/clinical diagnosis only. Bias from the selection of cases and controls was considered low if cases were recruited consecutively, intermediate if not clearly described and high when there was a reasonable probability that the sample was not representative for the population of interest. Bias due to missing outcome data was judged low if less than 20% of participants were lost to follow‐up among both cases and controls. Bias due to incomplete reporting was judged low if measurement methods, methods of analysis and outcomes were specified in advance. Bias due to unrepresentativeness of cases and controls was considered low when patients were recruited consecutively, medium when this was not clearly described and high when the sample did not seem representative. Handling complete outcome data was considered low when <10% of data were missing or sensitivity analyses or imputation were applied, medium when this was unclear or only complete case analysis was applied, and high when there was a significant amount of data missing that was likely not at random.

## RESULTS

**FIGURE 1:**
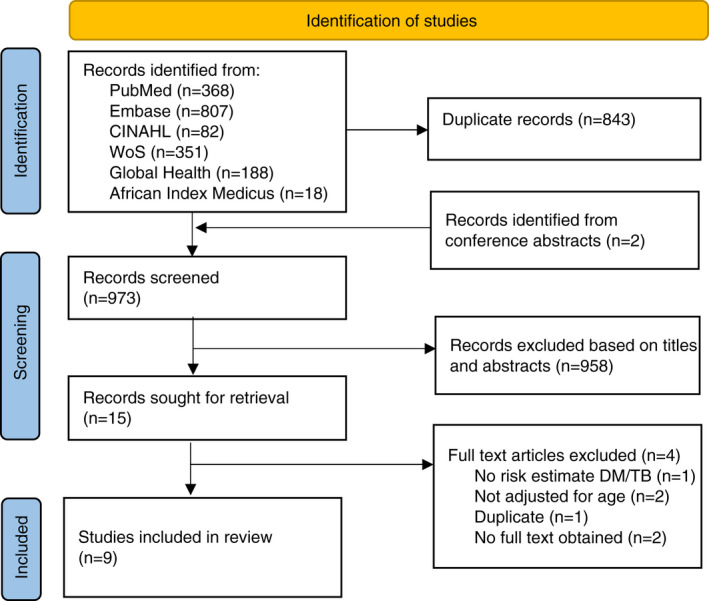
Flow diagram of the identification and selection of studies investigating the association between diabetes mellitus and tuberculosis in sub‐Saharan Africa

The database searches for studies identified 971 records after deduplication (Figure 1). Additionally, two papers were identified through screening of conference abstracts. No further studies were identified by screening reference lists of key reviews. Subsequently, 973 titles and abstracts were assessed, of which 958 were excluded because they did not meet the eligibility criteria. No studies were excluded because of the language requirements. For two studies, the full text could not be obtained. Consequently, 13 full texts were screened, of which, 9 studies were found to meet the eligibility criteria. Two study authors were contacted for clarification of data in their primary publication, neither of whom responded.

Individual study characteristics are presented in Table 1. All studies were carried out between 2009 and 2016. The publication dates ranged from 2011 [20] to 2020 [21]. Among the included studies, there were four case‐control studies [20, 22, 23, 24], one cohort study [21] and four cross‐sectional studies [25, 26, 27, 28]. The studies were conducted in various countries: two in South Africa [21, 25], one in Nigeria [26], three in Tanzania [20, 22, 23], one in Zambia [28], one in Guinea‐Bissau [24] and one in both South Africa and Zambia [27]. Four of the studies were conducted in hospital settings [21, 22, 26, 28], three in community settings [24, 25, 27] and one in both hospital and community settings [20]. Sample sizes ranged from 663 [26] to 90,601 [27]. The mean or median age ranged from 26.5 [24] to 38.5 [23]. All studies were conducted among both men and women (usually in similar proportions). The prevalence of HIV ranged from 5.2% in a Tanzanian study [23] to 67.3% in the study performed in hospitalised patients in Zambia [28].

**TABLE 1:** Study characteristics of the studies included determining the association between diabetes mellitus and tuberculosis in sub‐Saharan Africa

First author, (year)	Country, setting	Study period	Study design	Sample size	DM ascertainment	TB ascertainment	Primary comparison	Age mean/median (sd/IQR)	HIV+venumber (%)	Variables adjusted for
Kubjane et al. (2020) [21]	South Africa, hospital	July 2013 ‐ August 2015	Cohort	850	FBG ≥7 mmol/L, HbA1c ≥6.5% or self‐reported	Pulmonary TB, determined by GeneExpert	Patients presenting to the clinic with respiratory symptoms with a negative GeneExpert and resolution of symptoms within 3 months without TB treatment	38 (31–47)	519 (61.1)	Age, sex, HIV, hypertension, household size, income, previous miner, previous prisoner, marital status, work status
Sinha et al. (2018) [25]	South Africa, community	2010–2015 and 2015–2016	Cross‐sectional	7708	RBG >11.0 mmol/l or self‐reported	Pulmonary TB, determined by presence of one or more of the following TB symptoms: cough of any duration, fever of any duration, weight loss, night sweats	All participants without DM	42.6 (20.5)	837 (10.9)	Age, sex, HIV, receipt monthly grant, access to tap water, access to toilet, access to solar/electric energy
Lawson et al. (2017) [26]	Nigeria, hospital	NR	Cross‐sectional	663	HbA1c >6.4% or self‐reported in interview	Pulmonary TB, determined by sputum culture	Patients presenting to the clinic with presumptive TB (cough >2 weeks) without DM	37.8 (12.6)	184 (45.9)	Age, sex, HIV status
Boillat‐Blanco et al. (2016) [22]	Tanzania, hospital	June 2012 – December 2013	Case‐control	1035	Repeated measure FCG ≥7.0 mmol/l, OGTT ≥11.1 mmol/l, HbA1c ≥6.5% or history of and treatment for DM	New active TB, determined by sputum smear microscopy, chest radiography or clinical diagnosis	Sex and age‐matched controls selected from adults accompanying patients other than the included patients	36.3 (12.5)	232 (22.7)	Age, sex, BMI, HIV, socio‐economic status
Senkoro et al. (2016) [23]	Tanzania, setting not reported	NR	Case‐control	7163	Self‐reported	Pulmonary TB, confirmed with positive sputum culture or at least 2 smear positive results for AFB or one smear positive for AFB and chest X‐ray	All participants with presumptive TB who are bacteriologically negative and a random sample of people without presumptive TB	38.5 (17.5)	313 (5.2)	Age, sex, history of previous TB, BMI, HIV
Bailey et al. (2016) [27]	Zambia and South Africa, community	January 2010 – December 2010	Cross‐sectional	90,601	RBG >11 mmol/l	Pulmonary TB, determined by sputum culture, confirmed with RNA sequencing	All participants without DM	30^a^	6517 (7.2)	Age, sex, household economic position, education, BMI, HIV status, geographical location
Haraldsdottir et al. (2015) [24]	Guinea‐Bissau, community	July 2010 – July 2011	Case‐control	700	RBG ≥7 mmol/l at inclusion confirmed with 2 FBG >7 mmol/l or registered at DM clinic	Pulmonary TB, determined by sputum smear microscopy or chest radiography plus relevant, signs, symptoms and chest radiography changes after ineffective antibiotic treatment	Non‐TB controls, identified by random selection of houses in the study area	26.5^a^	NR	Age, sex, BMI
Bates et al. (2012) [28]	Zambia, hospital	September 2010 – December 2011	Cross‐sectional	964 (275 with NCD)	DM as admission diagnosis to hospital	Pulmonary TB, determined by sputum microscopy and culture	Participants with a NCD (except DM) as admission diagnosis	35 (28–43)	606 (67.3)	Age, HIV
Faurholt‐Jepsen et al. (2011) [20]	Tanzania, hospital and community	April 2006 – January 2009	Case‐control	1221	FBG > 6 mmol/L or OGTT > 11 mmol/L	Pulmonary TB, confirmed with sputum smear microscopy and sputum culture	Randomly selected sex and age‐matched controls living in same neighbourhood	34.3 (12.0)	382 (33.1)^a^	Age, sex, HIV, socio‐demography^b^

Abbreviations: DM, diabetes mellitus; TB, tuberculosis; FBG, fasting blood glucose; HbA1c, glycosylated haemoglobin; HIV, human immunodeficiency virus; RBG, random blood glucose; NR, not reported; FCG, fasting capillary glucose; OGTT, oral glucose tolerance test; BMI, body mass index; AFB, acid fast bacilli; NCD, non‐communicable disease.

^a^ The age mean/median was not reported by the study authors, but calculated by the researchers for the purpose of this review.

^b^ This study also presented a model which additionally adjusted for serum alpha‐1‐acid glycoprotein, because it was uncertain whether this was a confounder or whether it was on the pathway between DM and TB risk, the model that did not control for this was chosen.

Different methods of DM ascertainment were used and most studies used more than one method. Most studies asked about doctor diagnosed DM and also used fasting blood glucose (FBG), fasting capillary glucose (FCG), random blood glucose (RBG), the oral glucose tolerance test (OGTT) or measurement of HbA1c to identify people with undiagnosed DM. The study by Senkoro et al. [23] only used self‐reported DM status by patients and the study by Bates et al. [28] seemed to only report DM when this was the admission diagnosis to the hospital, although this was not clearly described. TB diagnosis was ascertained by sputum culture, sputum smear microscopy, chest radiography or nucleic acid amplification tests. The study by Sinha et al. [25] reported TB when a patient had one or more TB symptoms (cough, fever, night sweats or weight loss). As this was an inclusion criterion, all studies were adjusted for age. Seven studies [20, 21, 22, 23, 25, 26, 27] adjusted for sex and HIV. Other clinical factors adjusted for were BMI, hypertension and history of TB. Multiple studies also adjusted for socio‐economic factors, such as household size, marital status, access to tap water/energy and educational level. Four of the studies [20, 22, 24, 25] prespecified their set of confounders based on previous evidence, while four other studies [21, 23, 26, 27] used data‐driven methods to establish their set of confounders. For one study [28], the model used is unclear from the publication.

### Effect of DM on TB risk

**FIGURE 2:**
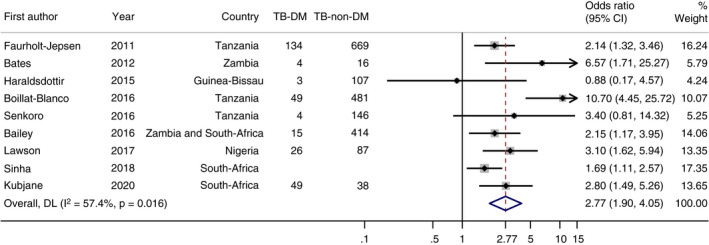
Forest plot of the meta‐analysis of the association between DM and TB in sub‐Saharan Africa

Individual study estimates are presented in Table 2. All studies reported an OR as the outcome measure for the association. Seven of the studies [20, 21, 22, 25, 26, 27, 28] found a statistically significant elevated risk of TB among people with DM. The ORs ranged from 0.88 (95% CI 0.17–4.58) [24] to 10.7 (95% CI 4.5–26) [22]. The pooled OR for the association between DM and TB was 2.77 (95% CI 1.90–4.05) (Figure 2). With the chi‐squared test, significant heterogeneity was identified (*I* ^2^ = 57%; *p* = 0.016).

**TABLE 2:** Individual study estimates of the unadjusted and adjusted odds ratios of active tuberculosis comparing DM prevalence in TB cases and non‐TB controls in sub‐Saharan Africa

First author, (year)	Method of DM diagnosis	Number (%) of TB cases with DM	Number (%) of non‐TB controls with DM	Unadjusted OR of active TB (95% CI)	Adjusted OR of active TB (95% CI)
Kubjane et al. (2020) [21]	FBG, HbA1c, self‐reported	At enrolment: 49 (11.9) After follow‐up: 28 (9.3)	38 (8.7) 27 (8.1)	Not reported	2.8 (1.5–5.3)^a^ 3.3 (1.5–7.3)
Sinha et al. (2018) [25]	RBG, self‐reported	>1 TB symptom^b^ >2 TB symptoms >3 TB symptoms	Not reported	Not reported	1.36 (1.11–1.67) 1.47 (1.13–1.91) 1.69 (1.11–2.57)^a^
Lawson et al. (2017) [26]	HbA1c, self‐reported	26 (23.0)	36 (12.1)	2.39 (1.35–4.24)	3.10 (1.62–5.94)^a^
Boillat‐Blanco et al. (2016) [22]	FCG OGTT HbA1c	24 (4.5) 36 (6.8) 49 (9.3)	6 (1.2) 15 (3.1) 11 (2.2)	4.2 (1.7–10.3) 2.9 (1.5–5.4) 6.5 (3.3–12.9)	10.6 (3.2–4.1)^c^ 3.7 (1.6–8.3) 10.7 (4.5–26)^a^
Senkoro et al. (2016) [23]	Self‐reported	4 (2)	45 (1)	3.1 (0.6–16.4)	3.4 (0.8–14.2)^a^
Bailey et al. (2016) [27]	RBG	15 (3.5)	712 (1.8)	Not reported	2.15 (1.17–3.94)^a^
Haraldsdottir et al. (2015) [24]	RBG, FBG, registered at DM clinic	3 (2.8)	11 (2.1)	Not reported	0.88 (0.17–4.58)^a^
Bates et al. (2012) [28]	DM as admission diagnosis to hospital	4 (20.0)	15 (5.9)	4.00 (1.19–13.5)	6.57 (1.71–25.30)^a^
Faurholt‐Jepsen et al. (2011) [20]	FBG and OGTT	134 (16.7)	33 (9.4)	2.2 (1.5–3.4)	HIV −: 2.14 (1.32–3.46)^a^ HIV +: 2.05 (0.68–6.29)

Abbreviations: DM, diabetes mellitus; TB, tuberculosis; OR, odds ratio; FBG, fasting blood glucose; HbA1c, glycosylated haemoglobin; RBG, random blood glucose; FCG, fasting capillary glucose; OGTT, oral glucose tolerance test.

^a^ These are the ORs that were included in the main meta‐analysis.

^b^ The number of cases and controls was not reported by the study authors.

^c^ This is an incorrect confidence interval that was reported by the study authors.

### Estimates stratified by HIV status

Four studies stratified their estimates by HIV status [20, 21, 22, 27] (Table 3). All studies showed a positive association between TB and DM among both people who were HIV‐positive and people who were HIV‐negative. In none of the studies, the difference between the two groups was statistically significant. However, in two studies [20, 21], the association appeared stronger in HIV‐negative in comparison with HIV‐positive. In one study [27] the association appeared weaker in HIV‐negative. In the study by Boillat‐Blanco et al. [22], the association appeared stronger in HIV‐positive for FCG and weaker for HbA1c. When the OGTT was applied, effect‐modification did not seem to occur. Interestingly, in the study by Faurholt‐Jepsen et al. [20], the difference became significantly larger when the association was adjusted for alpha‐1‐acid glycoprotein levels, a marker of inflammation.

**TABLE 3:** Individual study estimates of the adjusted odds ratios of active tuberculosis comparing DM prevalence in TB cases and non‐TB controls in sub‐Saharan Africa, stratified by HIV status

		HIV uninfected	HIV infected
First author, (year)	Method of DM diagnosis	Adjusted OR of active TB (95% CI)	Adjusted OR of active TB (95% CI)
Kubjane et al. (2020) [21]	FBG, HbA1c, self‐reported	3.5 (1.2–9.8)	2.4 (1.0–5.3)
Boillat‐Blanco et al. (2016) [22]	FCG OGTT HbA1c	8.8 (2.1–36.6) 3.8 (1.4–10.5) 19.3 (6.1–61.0)	17.1 (1.6–179.4) 3.8 (1.0–15.3) 4.7 (1.1–20.8)
Bailey et al. (2016) [27]	RBG	1.90 (0.89–4.04)	5.34 (1.56–18.23)
Faurholt‐Jepsen et al. (2011) [20]	FBG and OGTT	2.14 (1.32–3.46)^a^ 4.23 (1.54–11.57)^b^	2.05 (0.68–6.19)^a^ 0.14 (0.01–1.81)^b^

Abbreviations: DM, diabetes mellitus; TB, tuberculosis; FBG, fasting blood glucose; HbA1c, glycosylated haemoglobin; FCG, fasting capillary glucose; OGTT, oral glucose tolerance test; RBG, random blood glucose.

^a^ This estimate resulted from model 1 that adjusted for age, sex, HIV and socio‐demography.

^b^ This estimate resulted from model 2 that additionally adjusted for serum alpha‐1‐acid glycoprotein levels.

### Sensitivity analysis

The pooled OR did not change significantly in the sensitivity analyses with the alternative ORs from Kubjane's [21] study at follow‐up rather than commencement of TB treatment (2.84, 95% CI 1.92–4.20); Sinha et al. [25], for clinical TB diagnosis based on fewer TB symptoms, (for ≥1 symptoms 2.71, 95% CI 1.73–4.23 and for ≥2 symptoms 2.72, 95% CI 1.80–4.13) and Faurholt‐Jepsen et al. [20], for HIV‐positive participants, (2.85, 95% CI 1.88–4.32). However, in the study by Boillat‐Blanco et al. [22], when the OR for DM ascertainment by the OGTT was included instead of the estimate for HbA1c, the pooled OR was somewhat attenuated; 2.34 (95% CI 1.85–2.95), and statistical heterogeneity disappeared (*p* = 0.38; *I* ^2^ = 6.9%). A sensitivity analysis excluding the study by Senkoro et al. [23], which was the only study that used solely patient self‐report to identify people with DM, resulted in a pooled OR of 2.75 (95% CI 1.85–4.11). In a sensitivity analysis excluding the study by Sinha et al. [25], which diagnosed TB based on symptoms, the pooled OR was slightly higher at 3.08 (95% CI 2.036–4.648).

### Publication bias

**FIGURE 3:**
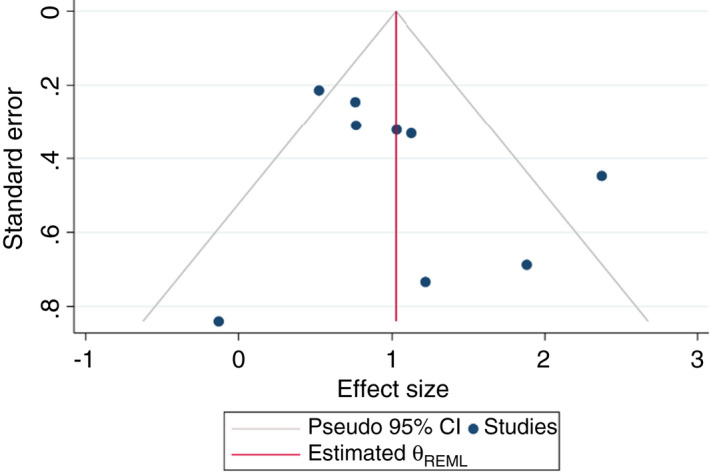
Funnel plot of the studies included investigating the association between DM and TB in sub‐Saharan Africa

The funnel plot appears broadly symmetric (Figure 3). Consequently, these is no evidence that publication bias has influenced the outcomes.

### Quality assessment

Individual study risk of bias is shown in Table 4. The definition of DM differed with some studies relying on self‐report or medical records, which might misclassify many undiagnosed DM patients as non‐DM. Three other studies [24, 25, 27] used random blood glucose measurement, which is not a recommended test to screen for DM [29], due to its low sensitivity [30]. The study by Sinha et al. [25] only used presence of TB symptoms to diagnose TB, which is not specific and potentially leads to misclassification [31]. Boillat‐Blanco et al. [22] also included clinical diagnosis of TB. One hospital‐based study selected [28] patients presenting to the hospital with DM as the admission diagnosis, and compared TB risk with control patients admitted with a different non‐communicable disease. By only selecting hospital cases, they likely included patients with more severe DM. In four studies [24, 25, 26, 27], the non‐response rate exceeded 20% due to difficulty obtaining cases and controls, and two studies [25, 28] did not report the non‐response rate. While in many studies, there was no description of handling incomplete outcome data, missing data was often <10%. As this was an inclusion criterion, all studies adjusted for age and also for other important confounders.

**TABLE 4:** Risk of bias of the studies that were included, assessed by the researchers

First author, (year)	Ascertainment DM	Ascertainment TB	Same ascertainment method cases and controls	Selection of cases/exposed	Selection of controls	Non‐response rate	Representativeness exposed/cases	Representativeness controls	Handling incomplete outcome data
Kubjane et al. (2020)^a^ [21]									
Sinha et al. (2018) [25]									
Lawson et al. (2017) [26]									
Boillat‐Blanco et al. (2016) [22]									
Senkoro et al. (2016) [23]									
Bailey et al. (2016) [27]									
Haraldsdottir et al. (2015) [24]									
Bates et al. (2012) [28]									
Faurholt‐Jepsen et al. (2011) [20]									

Green: low risk of bias, orange: medium risk of bias or unclear, red: high risk of bias.

^a^ This study was a cohort study with a follow‐up rate of 75% after 3 months of follow‐up.

## DISCUSSION

### Summary of findings

This systematic review investigated the association between DM and TB risk in SSA. Seven out of nine studies reported a significant elevated TB risk in DM patients. The pooled OR for the association was 2.77 (95% CI 1.90–4.05).

In line with evidence, the current review indicates a positive association between DM and TB. The review by Al‐Rifai et al., from 2017, identified a strong positive association, and meta‐analysis of the 44 studies included resulted in an overall OR of 2.00 (95% CI 1.78–2.24) [6]. Another systematic review, by Hayashi et al., from 2018, also found a positive relationship between DM and TB; the pooled OR was 1.50 (95% CI 1.28–1.76) [7]. However, both reviews identified significant heterogeneity between studies and only one African study was included [6, 7]. Contrary to our findings, a similar review on the association between DM and TB in SSA by Bailey et al., could not draw a conclusion on the presence of any association between TB and DM in SSA, because it included only three studies of which one showed a significant positive association and another one did not [13]. The authors concluded that the association between DM and TB may be different in an African population, possibly due to the high prevalence of HIV, poorer DM control and heterogeneity in DM phenotype and presentation [13].

The association in the current African specific review appears to be consistent with that identified in previous global reviews, or possibly even slightly stronger. An explanation could be that DM is often less well controlled in patients in SSA, or that the patients with TB‐DM recruited from SSA studies seemed to be younger than in previous reviews. Multiple studies have shown that poor glycaemic control is associated with a higher TB risk [10]. However, caution should be taken to conclude this based on the limited number of studies included.

Congruent with the previous review by Bailey et al., the current review could not draw a strong conclusion on the presence and magnitude of effect‐modification by HIV [13]. Two of the four studies included for this sub‐focus showed a stronger estimate in HIV‐negative, and one a weaker. In the last study, this depended on which diagnostic test was used for DM. However, the association between DM and TB appeared to be present in both HIV‐positive and negative people.

### Strengths and limitations of the review process

This review identified 9 eligible studies, compared with 3 studies in a former systematic review by Bailey et al. and therefore provides substantially more evidence [13]. One additional study from the period in which the former review searched was identified and the other 5 papers were published more recently. Furthermore, an extensive search was performed using a sensitive search strategy, built and translated in consultation with an information specialist, in six different databases, including one global health and one African specific database. We also searched references of multiple key reviews and identified 2 studies from checking conference abstracts. To increase the robustness of the review, titles and abstracts and full‐text screening, data extraction and quality assessment were performed by two researchers independently. Finally, possible reasons for heterogeneity were explored in sensitivity analyses, which showed that heterogeneity was driven mainly by one specific estimate from a single study.

A limitation of the review process is that the search for grey literature was restricted to screening conference abstracts. Additionally, only studies in English and French were included, but it is unlikely that this had an impact, since no studies were excluded because of this language restriction. However, studies not indexed in the main medical databases, for example, grey literature in local languages, might have been missed by the search strategy. A number of studies were excluded due to lack of adjustment for age. However, studies that did not adjust for age mostly only reported unadjusted odds ratios, which could be biased.

### Strengths and limitations of the studies included

The most important limitations of the studies included concerned DM ascertainment, which was not always performed according to international guidelines. For example, the majority of the studies based on DM diagnosis, either on patient self‐report, or through only a single blood glucose or HbA1c measurement. Self‐report is clearly insensitive in resource poor settings. However, when the one study that used only self‐report as a measure for DM was excluded in a sensitivity analysis, the pooled OR did not change significantly. WHO recommends repeated blood glucose or HbA1c measurements at different time points to diagnose DM in those without classical DM symptoms [32]. Since TB disease can result in hyperglycaemia, repeated measurements might be of even greater importance [33]. The difference in diagnostic tests and cut‐points used between studies also makes it difficult to compare the study outcomes and might lead to heterogeneity between studies.

A strength of the included studies was that they all adjusted for age and other important confounders, such as sex and HIV status. Four of the studies additionally adjusted for socio‐economic status, by measuring related factors, such as income, access to energy and educational level. Since many factors related to a low socio‐economic status, such as poor housing and crowded living conditions, are established risk factors for TB and DM, these studies may report more reliable results [32]. No evidence of publication bias was found, which could be explained by the small body of literature available from SSA on the association, which makes it more likely that small studies with insignificant outcomes will be published. However, with only a small number of studies included, such conclusions should be interpreted with caution.

### Implications

This review implies that people with DM in SSA have an almost three times higher risk to be diagnosed with active TB disease than people without DM. While HIV is still the strongest risk factor for TB in SSA, DM likely also contributes to TB epidemiology [12]. Since the prevalence of DM is increasing rapidly in SSA, this population effect is expected to become more profound over the next few decades. Currently, SSA is making good progress to reach the TB incidence milestone of the WHO’s end TB strategy, which is to reduce TB incidence rates by 80% by 2030 in comparison with 2015 [1]. However, the increasing prevalence of DM could potentially affect the fast decline and threaten reaching the WHO’s end TB targets in SSA.

The very young mean age of patients in the studies included was notable. Comorbidity of DM and HIV will likely become more prevalent as cohorts of people with HIV start to age in SSA. In addition, people with HIV have a higher risk of contracting DM, due to ART treatment [34]. Because this and former reviews could not draw strong conclusions on the presence and magnitude of effect‐modification by HIV, a key evidence gap remains whether HIV status modifies the association between DM and TB. To address this, more studies on the association between DM and TB that stratify by HIV status should be performed. These studies should have a larger number of participants, so that stratifying is justified, and may thus require the use of routine records, historically difficult in SSA. Importantly, these studies should specify whether HIV is treated or untreated and early or advanced, because these factors could potentially influence the association.

## CONCLUSIONS

This systematic review implies that people with DM in SSA have an almost three times higher risk of developing active TB, in accordance with evidence from other continents. However, more research is needed to determine whether and how HIV modifies this relationship, in order to fully understand the potential future impact of rising DM prevalence on TB epidemics in SSA.

## Figures and Tables

**FIGURE A1:**
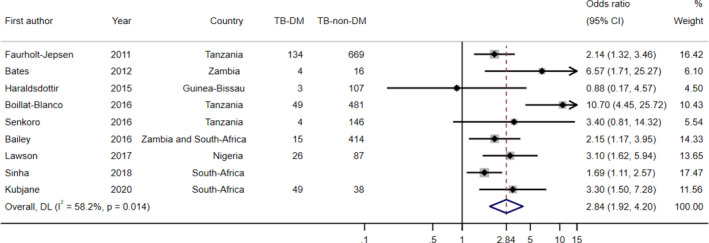
Sensitivity analysis including the OR at follow‐up of the study of Kubjane et al.

**FIGURE A2:**
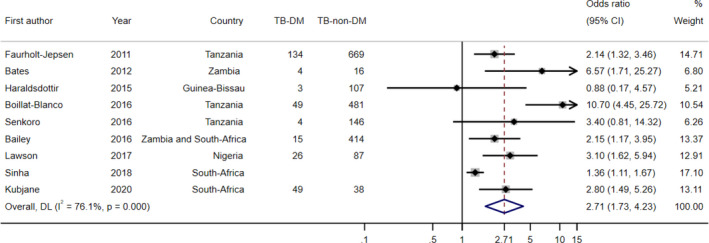
Sensitivity analysis including the OR for ≥1 TB symptoms of the study of Sinha et al.

**FIGURE A3:**
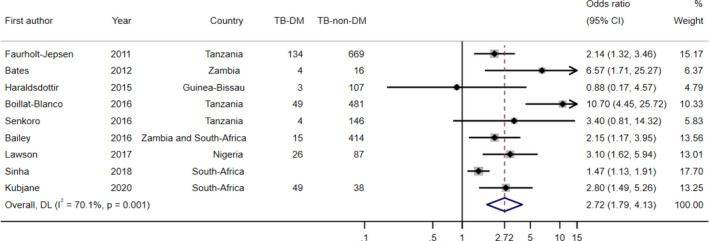
Sensitivity analysis including the OR for ≥2 TB symptoms of the study of Sinha et al.

**FIGURE A4:**
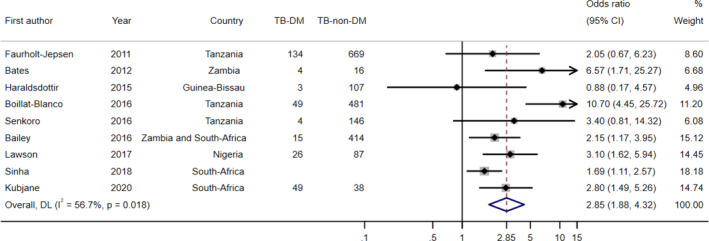
Sensitivity analysis including the OR for HIV‐positive participants of the study by Faurholt‐Jepsen et al.

**FIGURE A5:**
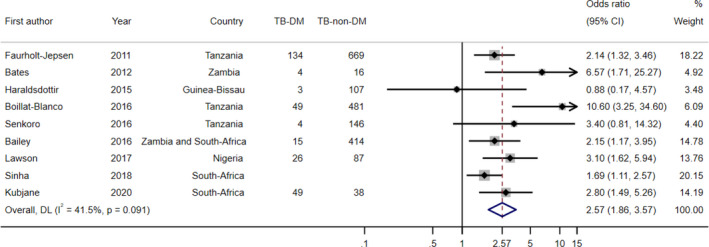
Sensitivity analysis including the OR for FCG measurement of the study by Boillat‐Blanco et al.

**FIGURE A6:**
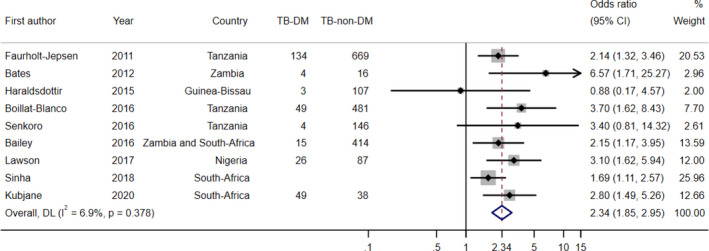
Sensitivity analysis including the OR for the OGTT of the study by Boillat‐Blanco et al.

**FIGURE A7:**
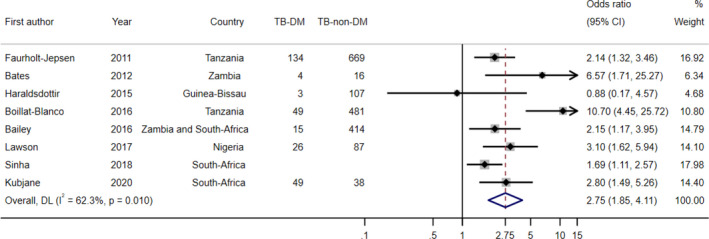
Sensitivity analysis removing the estimate from the study by Senkoro et al.

**FIGURE A8:**
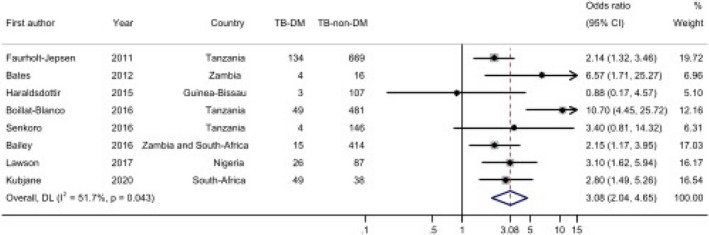
Sensitivity analysis removing the estimate from the study of Sinha et al.

## APPENDIX 1

### SEARCH STRATEGY

The search strategy below is the exact search term that was applied in PubMed. For searches in the other databases, the search strategy was translated. The searches were performed on the 21st of February 2021. We searched from January 1980, without language restrictions.

("Tuberculosis"[Mesh] OR tuberculo*[tiab] OR TB[tiab] OR koch disease*[tiab] OR koch's disease*[tiab])

AND

("Diabetes Mellitus"[Mesh] OR "Hyperglycemia"[Mesh] OR diabet*[tiab] OR Hyperglycaemi*[tiab] OR Hyper‐glycemi*[tiab] OR Dysglycaemi*[tiab] OR Dysglycemi*[tiab] OR Disglycemi*[tiab] or disglycaemi*[tiab])

AND

("Africa"[Mesh] OR Africa[tiab] OR Cameroon[tiab] OR Central African Republic[tiab] OR Chad[tiab] OR Congo[tiab] OR Democratic Republic of the Congo[tiab] OR Equatorial Guinea[tiab] OR Gabon[tiab] OR Sao Tome[tiab] OR Burundi[tiab] OR Djibouti[tiab] OR Eritrea[tiab] OR Ethiopia[tiab] OR Kenya[tiab] OR Rwanda[tiab] OR Somalia[tiab] OR South Sudan[tiab] OR Sudan[tiab] OR Tanzania[tiab] OR Uganda[tiab] OR Angola[tiab] OR Botswana[tiab] OR Eswatini[tiab] OR Lesotho[tiab] OR Malawi[tiab] OR Mozambique[tiab] OR Namibia[tiab] OR South Africa[tiab] OR Zambia[tiab] OR Zimbabwe[tiab] OR Benin[tiab] OR Burkina Faso[tiab] OR Cabo Verde[tiab] OR Cote d'Ivoire[tiab] OR Ivory coast[tiab] OR Gambia[tiab] OR Ghana[tiab] OR Guinea[tiab] OR Guinea‐Bissau[tiab] OR Liberia[tiab] OR Mali[tiab] OR Mauritania[tiab] OR Niger[tiab] OR Nigeria[tiab] OR Senegal[tiab] OR Sierra Leone[tiab] OR Togo[tiab] OR Madagascar[tiab] OR Pemba[tiab] OR Zanzibar[tiab] OR Comoros[tiab] OR Seychelles[tiab] OR Mauritius[tiab])

## APPENDIX 2

### QUALITY ASSESSMENT

This appendix contains the tools applied for quality assessment and the scores assigned to the studies per question by the researchers. For the studies with a cross‐sectional design, the quality assessment tool for observational, cohort and cross‐sectional studies of the National Institute of Health was applied [30] (Table A1). For case‐control studies, the Newcastle‐Ottawa scale for case‐control studies [29] was used (Table A2) and for cohort studies, the Newcastle‐Ottawa scale for cohort studies was used [29] (Table A3). The exact questions of each scale can be found in this appendix after the tables with the scores assigned by the researchers.

**TABLE A1:** Risk of bias assessment^a^ of the studies with a cross‐sectional design

Author, year	Research question clear	Clearly defined study population	Participation rate >50%	Participants from same population/time and eligibility criteria uniformly applied	Sample size justification	Exposure assessed prior to outcome	Timeframe sufficient to detect exposure and outcome	Different levels of exposure assessed	Exposure measure clearly defined, valid, reliable, applied consistently	Exposure measure assessed more than once	Outcome measures clearly defined, valid, reliable and applied consistently	Outcome assessors blinded to exposure	Key confounders measured and adjusted for
Sinha et al. (2018)	Yes	Yes	No	Yes	No	No	No	No	No	No	No	?	Yes
Lawson et al. (2017)	Yes	No	Yes	Yes	No	No	No	Yes	Yes	No	Yes	?	Yes
Bailey et al. (2016)	Yes	Yes	Yes	Yes	No	No	No	Yes	Yes	No	Yes	?	Yes
Bates et al. (2012)	Yes	Yes	Yes	Yes	No	No	No	No	No	No	Yes	?	Yes

^a^ Quality assessment was performed using the Quality assessment tool for observational, cohort and cross‐sectional studies of the National Institute of Health. Questions are answered with yes or no. When it was unclear, a question mark was reported.

**TABLE A2:** Risk of bias assessment^a^ of the studies with case‐control design

	Selection					Outcome			
Author, year	Adequacy case definition	Representativeness cases	Selection of controls	Definition of controls	Comparability of cases and controls on the basis of design or analysis	Ascertainment of exposure	Same method ascertainment cases and controls	Non‐response rate	Final score
Boillat‐Blanco et al. (2016)	1	1	1	1	2	1	1	0	8
Senkoro et al. (2016)	1	1	1	1	2	0	1	0	7
Haraldsdottir et al. (2015)	1	1	1	1	2	1	1	0	8
Faurholt‐Jepsen et al. (2011)	1	1	1	1	2	1	1	1	9

^a^ Quality assessment was performed using the Newcastle‐Ottawa scale for case‐control studies. For the questions concerning selection and outcome, 1 point is assigned when adequate and 0 points if not. For the question on comparability of cohorts, a maximum of 2 points can be assigned.

**TABLE A3:** Risk of bias assessment^a^ of the study with a cohort design

	Selection					Outcome			
Author, year	Representativeness of the exposed cohort	Selection of the non‐exposed cohort	Ascertainment of exposure	Demonstration that outcome of interest was not present at start of study	Comparability of cohorts on the basis of the design or analysis	Assessment of outcome	Was follow‐up long enough for outcomes to occur	Adequacy of follow‐up of cohorts	Final score
Kubjane et al. (2020)	1	1	1	0	2	1	1	0	7

^a^ Quality assessment was performed using the Newcastle‐Ottawa scale for cohort studies. For the questions concerning selection and outcome 1 point is assigned when adequate and 0 points if not. For the question on comparability of cohorts a maximum of 2 points can be assigned.

### QUALITY ASSESSMENT TOOL FOR OBSERVATIONAL, COHORT AND CROSS‐SECTIONAL STUDIES OF THE NATIONAL INSTITUTE OF HEALTH [30]

1. Was the research question or objective in this paper clearly stated?
2. Was the study population clearly specified and defined?
3. Was the participation rate of eligible persons at least 50%?
4. Were all the subjects selected or recruited from the same or similar populations (including the same time period)? Were inclusion and exclusion criteria for being in the study prespecified and applied uniformly to all participants?
5. Was a sample size justification, power description, or variance and effect estimates provided?
6. For the analyses in this paper, were the exposure(s) of interest measured prior to the outcome(s) being measured?
7. Was the timeframe sufficient so that one could reasonably expect to see an association between exposure and outcome if it existed?
8. For exposures that can vary in amount or level, did the study examine different levels of the exposure as related to the outcome (e.g., categories of exposure, or exposure measured as continuous variable)?
9. Were the exposure measures (independent variables) clearly defined, valid, reliable and implemented consistently across all study participants?
10. Was the exposure(s) assessed more than once over time?
11. Were the outcome measures (dependent variables) clearly defined, valid, reliable and implemented consistently across all study participants?
12. Were the outcome assessors blinded to the exposure status of participants?
13. Was loss to follow‐up after baseline 20% or less?
14. Were key potential confounding variables measured and adjusted statistically for their impact on the relationship between exposure(s) and outcome(s)?

### **NEWCASTLE‐OTTAWA SCALE FOR CASE‐CONTROL STUDIES** [29]

Items are rewarded a point when answer the with the * applies.

#### Selection

1. Is the Case Definition Adequate?
  - Requires some independent validation (e.g. >1 person/record/time/process to extract information, or reference to primary record source such as x‐rays or medical/hospital records.*
  - Record linkage (e.g. ICD codes in database) or self‐report with no reference to primary record.
  - No description.
2. Representativeness of the Cases
  - All eligible cases with outcome of interest over a defined period of time, all cases in a defined catchment area, all cases in a defined hospital or clinic, group of hospitals, health maintenance organisation, or an appropriate sample of those cases (e.g. random sample)*.
  - Not satisfying requirements in part (a), or not stated.
3. Selection of Controls
  This item assesses whether the control series used in the study is derived from the same population as the cases and essentially would have been cases had the outcome been present.
  - Community controls (i.e. same community as cases and would be cases if had outcome)*.
  - Hospital controls, within same community as cases (i.e. not another city) but derived from a hospitalised population.
  - No description.
4. Definition of Controls
  - If cases are first occurrence of outcome, then it must explicitly state that controls have no history of this outcome. If cases have new (not necessarily first) occurrence of outcome, then controls with previous occurrences of outcome of interest should not be excluded.*
  - No mention of history of outcome.

#### Comparability

1. Comparability of Cases and Controls on the Basis of the Design or Analysis

A maximum of 2 stars can be allotted in this category.

Either cases and controls must be matched in the design and/or confounders must be adjusted for in the analysis. Statements of no differences between groups or that differences were not statistically significant are not sufficient for establishing comparability. Note: If the odds ratio for the exposure of interest is adjusted for the confounders listed, then the groups will be considered to be comparable on each variable used in the adjustment.

There may be multiple ratings for this item for different categories of exposure (e.g. ever vs. never, current vs. previous or never).

Age = *, Other controlled factors = *.

#### Exposure

1. Ascertainment of Exposure
  - secure record (eg surgical records)*
  - structured interview where blind to case/control status*
  - interview not blinded to case/control status
  - written self‐report or medical record only
  - no description
2. Same method of ascertainment for cases and controls
  - Yes*
  - No
3. Non‐Response Rate
  - same rate for both groups*
  - non‐respondents described
  - rate different and no designation

### NEWCASTLE‐OTTAWA SCALE FOR COHORT STUDIES [29]

Items are rewarded a point when answer the with the * applies.

#### Selection

1. Representativeness of the Exposed Cohort
  - truly representative of the average _______________ (describe) in the community*
  - somewhat representative of the average ______________ in the community*
  - selected group of users e.g. nurses, volunteers
  - no description of the derivation of the cohort
2. Selection of the Non‐Exposed Cohort
  - drawn from the same community as the exposed cohort*
  - drawn from a different source
  - no description of the derivation of the non‐exposed cohort
3. Ascertainment of Exposure
  - secure record (e.g. surgical records)*
  - structured interview*
  - written self‐report
  - no description
4. Demonstration That Outcome of Interest Was Not Present at Start of Study
  - Yes*
  - No

#### Comparability

1. Comparability of Cohorts on the Basis of the Design or Analysis

A maximum of 2 stars can be allotted in this category.

Either exposed and non‐exposed individuals must be matched in the design and/or confounders must be adjusted for in the analysis. Statements of no differences between groups or that differences were not statistically significant are not sufficient for establishing comparability. Note: If the relative risk for the exposure of interest is adjusted for the confounders listed, then the groups will be considered to be comparable on each variable used in the adjustment.

There may be multiple ratings for this item for different categories of exposure (e.g. ever vs. never, current vs. previous or never).

Age = *, Other controlled factors = *.

#### Outcome

1. Assessment of Outcome
  - Independent or blind assessment stated in the paper, or confirmation of the outcome by reference to secure records (x‐rays, medical records, etc.)*.
  - Record linkage (e.g. identified through ICD codes on database records)*.
  - Self‐report (i.e. no reference to original medical records or x‐rays to confirm the outcome).
  - No description.
2. Was Follow‐Up Long Enough for Outcomes to Occur
  - yes (select an adequate follow‐up period for outcome of interest)*.
  - no.
3. Adequacy of Follow‐Up of Cohorts
  - Complete follow‐up ‐ all subjects accounted for*.
  - Subjects lost to follow‐up unlikely to introduce bias ‐ small number lost >80% (select an follow‐up, or description provided of those lost)*.
  - Follow‐up rate <80% and no description of those lost.
  - No statement

## APPENDIX 3

### SENSITIVITY ANALYSIS

Because multiple studies did not report one overall estimate for the association between diabetes mellitus and tuberculosis, but several ORs for different tests or subgroups, a sensitivity analysis was performed with the estimates that were not included in the main meta‐analysis.

### SENSITIVITY ANALYSIS KUBJANE ET AL.

The study by Kubjane et al. reported two separate odds ratios (ORs), one for the association at enrolment of participants and one at follow‐up. In the initial meta‐analysis, the OR at enrolment was included, for consistency with other studies. A sensitivity analysis was performed including the OR at follow‐up (Figure A1). This resulted in a pooled OR of 2.84 (95% CI 1.92–4.20) and significant heterogeneity (*p* = 0.014, *I* ^2^ = 58.2%).

### SENSITIVITY ANALYSIS SINHA ET AL.

The study of Sinha et al., which ascertained TB as having TB symptoms, reported three different ORs; one OR for having ≥1 TB symptoms, one for having ≥2 TB symptoms and for having ≥3 TB symptoms. In the initial meta‐analysis, the OR for having 3 or more TB symptoms was included, because this is most specific for having active TB. Sensitivity analyses were performed with the other two estimates. The analysis including the OR for ≥1 symptoms resulted in a pooled OR of 2.71 (95% CI 1.73–4.23) and a significant level of heterogeneity (*p* = 0.00, *I* ^2^ = 76.1) (Figure A2). When the OR for having ≥2 TB symptoms was included, the pooled OR was 2.72 (95% CI 1.79–4.13) and again there was significant heterogeneity (*p* = 0.001, *I* ^2^ = 70.1%) (Figure A3).

### SENSITIVITY ANALYSIS FAURHOLT‐JEPSEN ET AL.

Faurholt‐Jepsen et al. reported the ORs for TB risk stratified by HIV status. Since the aim of the main meta‐analysis was not to assess the effect of HIV on the association between DM and TB, the OR for HIV‐negative subjects was included. When, for the purpose of the sensitivity analysis, the OR for HIV‐positive subjects was included, the pooled OR was 2.85 (95% CI 1.88–4.32) and heterogeneity was significant (*p* = 0.018, *I* ^2^ = 56.7%) (Figure A4).

### SENSITIVITY ANALYSIS BOILLAT‐BLANCO ET AL.

Boillat‐Blanco et al. used three different tests for DM ascertainment; FCG, the OGTT and HbA1c. They reported ORs for the association for each DM test separately. In the main meta‐analysis, the estimate using HbA1c measurement was included, because this was considered the most reliable of the three tests. A sensitivity analysis was performed for the two other estimates. When the FCG estimate was included, the pooled OR was 2.57 (95% CI 1.86–3.57) and heterogeneity was not significant (*p* = 0.091, *I* ^2^ = 31.5%) (Figure A5). When the OGTT estimate was included, the pooled OR was 2.34 (95% CI 1.85–2.95) and the level of heterogeneity was low (*p* = 0.378, *I* ^2^ = 6.9%) (Figure A6).

### SENSITIVITY ANALYSIS WITHOUT SENKORO ET AL.

The study by Senkoro et al. only used self‐report as a method to diagnose DM. In a sensitivity analysis, the estimate of this study was removed from the analysis. Removing this estimate resulted in an OR of 2.75 (95% CI 1.85–4.11) and the level of heterogeneity remained high (*p* = 0.010, *I* ^2^ = 62.3%) (Figure A7).

### SENSITIVITY ANALYSIS WITHOUT SINHA ET AL.

The study by Sinha et al. based TB diagnosis on symptoms, which is not a reliable method to diagnose TB. Therefore, the estimate of the study by Sinha et al. was removed in a sensitivity analysis, which resulted in an OR of 3.08 (95% CI 2.04–4.65) (Figure A8). Heterogeneity remained high (*p* = 0.043, *I* ^2^ = 51.7%).
